# Effects of the Proprioceptive Neuromuscular Facilitation Contraction Sequence on Motor Skill Learning-Related Increases in the Maximal Rate of Wrist Flexion Torque Development

**DOI:** 10.3389/fnhum.2021.764660

**Published:** 2021-11-03

**Authors:** Lara A. Green, Jessica McGuire, David A. Gabriel

**Affiliations:** ^1^Electromyographic Kinesiology Laboratory, Faculty of Applied Health Sciences, Brock University, St. Catharines, ON, Canada; ^2^Exercise Neuroscience Laboratory, Department of Kinesiology, Wilfrid Laurier University, Waterloo, ON, Canada

**Keywords:** muscle mechanics, motor learning, PNF, electromyography, flexor carpi radialis

## Abstract

**Background:** The proprioceptive neuromuscular facilitation (PNF) reciprocal contraction pattern has the potential to increase the maximum rate of torque development. However, it is a more complex resistive exercise task and may interfere with improvements in the maximum rate of torque development due to motor skill learning, as observed for unidirectional contractions. The purpose of this study was to examine the cost-benefit of using the PNF exercise technique to increase the maximum rate of torque development.

**Methods:** Twenty-six participants completed isometric maximal extension-to-flexion (experimental PNF group) or flexion-only (control group) contractions at the wrist. Ten of the assigned contractions were performed on each of three sessions separated by 48-h for skill acquisition. Retention was assessed with 5 contractions performed 2-weeks after acquisition. Torque and surface electromyographic (sEMG) activity were analyzed for evidence of facilitated contractions between groups, as well as alterations in muscle coordination assessed across test sessions. The criterion measures were: mean maximal isometric wrist flexion toque; the maximal rate of torque development (*d*τ/*dt*_*max*_); root-mean-square error (RMSE) variability of the rate of torque versus torque phase-plane; the rate of wrist flexion muscle activation (*Q*_30_); a coactivation ratio for wrist flexor and extensor sEMG activity; and wrist flexor electromechanical delay (EMD).

**Results:** There were no significant differences between groups with respect to maximal wrist flexion torque, *d*τ/*dt*_*max*_ or RMSE variability of torque trajectories. Both groups exhibited a progressive increase in maximal strength (+23.35% *p* < 0.01, *η*^2^ = 0.655) and in *d*τ/*dt*_*max*_ (+19.84% *p* = 0.08, *η*^2^ = 0.150) from the start of acquisition to retention. RMSE was lowest after a 2-week rest interval (−18.2% *p* = 0.04, *η*^2^ = 0.198). There were no significant differences between groups in the rate of muscle activation or the coactivation ratio. There was a reduction in coactivation that was retained after a 2-week rest interval (−32.60%, *p* = 0.02, *η*^2^ = 0.266). Alternatively, EMD was significantly greater in the experimental group (Δ 77.43%, *p <* 0.01, *η*^2^ = 0.809) across all sessions. However, both groups had a similar pattern of improvement to the third consecutive day of testing (−16.82%, *p* = 0.049, *η*^2^ = 0.189), but returned close to baseline value after the 2-week rest interval.

**Discussion:** The wrist extension-to-flexion contraction pattern did not result in a greater maximal rate of torque development than simple contractions of the wrist flexors. There was no difference between groups with respect to motor skill learning. The main adaptation in neuromotor control was a decrease in coactivation, not the maximal rate of muscle activation.

## Introduction

The resistive exercise literature has, almost in its entirety, been focused on the neural and hypertrophic mechanisms underlying training-related increases in maximum strength, while motor learning has received much less attention (Gabriel et al., 2006). The expression of muscle strength involves a skill component where repeated execution of the task results in motor learning of how to effectively activate agonists, synergists, and antagonists to produce the greatest joint torque (Kroll, 1981). Because the expression of muscle strength involves motor skill, it is affected by those factors that optimize task learning (Smith, 1974). For example, massed practice is superior to distributed practice when maximal effort contractions are involved. Massed isometric contractions of the elbow flexors allow study participants to better integrate proprioceptive feedback to update and refine the internal model of performance with each successive contraction, compared to distributed contractions (Calder and Gabriel, 2007). Surface electromyography (sEMG) revealed that participants who performed a massed contraction pattern exhibited a progressive decrease in antagonist muscle coactivation compared to contractions distributed over multiple days.

Resistance exercise task complexity is another motor learning variable that has received limited attention. Some resistive exercise machines have incorporated reciprocal concentric contractions, to enhance muscle activation through proprioceptive neuromuscular facilitation (Roy et al., 1990). However, Kroll (1972) demonstrated that resistance exercise of both agonist and antagonist muscle groups interfered with increases in maximal isometric strength due to task learning. Gabriel and Kroll (1991) then evaluated the cost-benefit of increasing task complexity to elicit proprioceptive neuromuscular facilitation (PNF) during the maximal isometric elbow extension-to-flexion contraction sequence. Not only did the extension-to-flexion contraction sequence fail to elicit PNF (strength and sEMG), but it also interfered with strength gains due to motor learning.

The cost-benefit of increasing task complexity to elicit PNF was later re-examined on the basis of utilizing the strength advantage of the elbow flexors to facilitate the weaker elbow extensors (Kroll et al., 1990; Gabriel et al., 1997). Gabriel et al. (1997) showed that the PNF contraction sequence did not interfere with motor learning-related strength gains. The experimental group (flexion-to-extension) and control group (extension-only) exhibited the same progressive increase in maximal isometric elbow extension strength across test sessions. However, the flexion-to-extension contraction pattern failed to elicit PNF in the extensors of the experimental group. Interestingly, the PNF sequence did result in significantly greater rates of torque development, without any observable difference in the magnitude of sEMG activity of the elbow extensors (Gabriel et al., 2001a). The lack of increase in sEMG magnitude, led the authors to suggest that the facilitated contractions may be due to musculoskeletal biomechanics: that is, during the flexion-to-extension sequence, the contracting flexors lengthen the extensors while coactive as antagonists, placing them at optimal muscle length immediately prior to their voluntary activation as an agonist (Gabriel et al., 2001a). Unfortunately, coactivation was not assessed as a probable mechanism in this particular study, but was later identified as a torque contributor by Richartz et al. (2010).

Richartz et al. (2010) studied the maximal rate of isometric dorsiflexion torque development in response to the following conditions: (1) relaxation of the dorsiflexors; (2) pre-activation of the dorsiflexors at 20% MVC; and (3) a rapid reversal contraction of the plantar flexors at 25, 50, and 75% of MVC. The average sEMG amplitude was calculated from its onset to 25 ms, 50 ms, and to the time point where *d*τ/*dt*_*max*_ occurred. The sEMG amplitude was comparable between the pre-activation and rapid reversal conditions, and both resulted in greater sEMG magnitude than initiating maximal isometric dorsiflexion from a complete rest. Despite having comparable sEMG magnitude to the pre-activation condition, the rapid reversal resulted in a markedly greater maximal rate of torque development. This suggested that the rapid reversal technique did more than eliminate slack in the series elastic component of the dorsiflexors. In agreement with Gabriel et al. (2001a) and Richartz et al. (2010) concluded that the contraction of plantar flexors lengthen the dorsiflexors while coactive as antagonists, placing them at optimal muscle length immediately prior to their voluntary activation as an agonist.

It is also possible that PNF may be manifested through the rate of increase of muscle activity, which was not assessed in the two previous studies (Gabriel et al., 2001a; Richartz et al., 2010). Kamimura et al. (2009) studied the PNF contraction sequence (extension-to-flexion) for the elbow flexors. There was a greater rate of elbow flexion torque and rate of rise in biceps brachii sEMG, compared to simple contractions of elbow flexors. Indirect support for a probable mechanism is given by Shimura and Kasai (2002). The authors examined the effect of PNF on sEMG, as induced via posture. The differences in sEMG onset of the brachioradialis, triceps brachii and medial deltoid were examined while participants performed a wrist extension reaction time task, with the upper limb in a neutral versus PNF posture (Shimura and Kasai, 2002). There was decreased sEMG latency for all muscles relative to the onset of the reaction stimulus. The decrease in sEMG onset is consistent with subthreshold changes in excitability that would lead to an increase in the rate of motor unit recruitment (Hayes, 1972; Moore and Kukulka, 1991; Moritani and Shibata, 1994; Harwood and Rice, 2012; Del Vecchio et al., 2019b). In support, Richartz et al. (2010) only found significantly greater activation from the rapid reversal contraction, compared to the pre-activation contractions, in the first 25 ms of activity. The first 25 ms period is notable because it is similar to the slope (*Q*_30_)of the sEMG signal as measured by integrating the signal for the first 30 ms (Gottlieb et al., 1989; Gabriel and Boucher, 2000).

The PNF contraction sequence is potentially a useful resistive exercise technique for increasing the maximum rate of torque development. However, it is not known if the underlying mechanism involves neuromuscular responses or musculoskeletal biomechanics. It is also important to establish that the PNF contraction pattern does not interfere with motor learning-related increases in the maximum rate of torque development and maximal torque levels. To this end, wrist flexion torque and sEMG of the flexor carpi radialis and extensor carpi radialis were monitored during experimental (extension-to-flexion) and control (flexion-only) contractions performed on three consecutive test sessions (acquisition phase) and repeated 2-weeks later (retention). The torque and sEMG data were analyzed for evidence of facilitated contractions between groups, as well as alterations in muscle coordination assessed across test sessions.

## Materials and Methods

Twenty-six male undergraduate kinesiology students (18–25 years old) participated in the study. They were free from neurological or musculoskeletal disorders of the upper limb, right-hand dominant. Although they were recreationally active, participants had not performed any forearm resistance training for at least 1 year prior. All participants completed written informed consent forms as approved by Brock University Research Ethics Board (REB#12-281).

### Apparatus and Testing Position

All procedures took place inside a Faraday cage within the Electromyographic Kinesiology Laboratory at Brock University. Participants were seated at a testing table so that the elbow could be placed at 160° of extension, while the forearm rested on the table and the hand was secured in a custom jig designed for isometric wrist flexion and extension contractions (Green et al., 2015). The forearm was half-supinated with hand restraints mounted onto a lever arm attached to a load cell (JR3 Inc., Woodland, CA, United States) that contacted the volar and dorsal surfaces. The axis of rotation of the wrist was aligned with the axis of rotation of the lever arm on the load cell. An oscilloscope (VC-6525, Hitachi, Woodbury, NY, United States) was placed at eye level in front of the participant to display the torque during contractions.

### Measurement Schedule

There was a preliminary session where participants reported to the laboratory to become familiarized with the testing environment and equipment. Anthropometric measurements were obtained for use in a multiple regression equation that included body weight, forearm length and elbow circumference to create a control group (*N* = 13) and experimental group (*N* = 13) that were matched on predicted maximal isometric wrist flexion strength. The purpose of creating matched groups from predicted strength, was to record the first attempts at task learning, without the previous familiarization that normally occurs with resistive exercise studies. This allowed alterations in neuromotor control to be monitored during the initial phases of motor skill learning. The control group performed maximal isometric contractions of the wrist flexors. Each contraction was 5 s in duration to allow participants to maintain a constant level of torque during the plateau; with 3-min of rest between each contraction to minimize fatigue (Clarke and Alan Stull, 1969). The experimental group first performed a 5 s maximal isometric contraction of the wrist extensors. At the end of 5 s, participants immediately reversed the direction of wrist torque to initiate a 5 s maximal isometric contraction of the wrist flexors. There was then 3 min of rest between each extension-to-flexion dyad.

Both groups were tested using a measurement schedule previously demonstrated to result in a progressive increase in maximal isometric strength of the wrist flexors due to motor skill learning (Kroll, 1963). The first three sessions were separated by 48 h each (acquisition phase), and the fourth session occurred 2-weeks after the third session (retention). At each session participants performed ten trials of their assigned contraction pattern. The number of trials was based on previous work that showed that changes in the variability of torque- and linear envelope detected sEMG-time curves plateaued by the tenth trial (Green et al., 2014).

### Instructions to Participants

**FIGURE 1:**
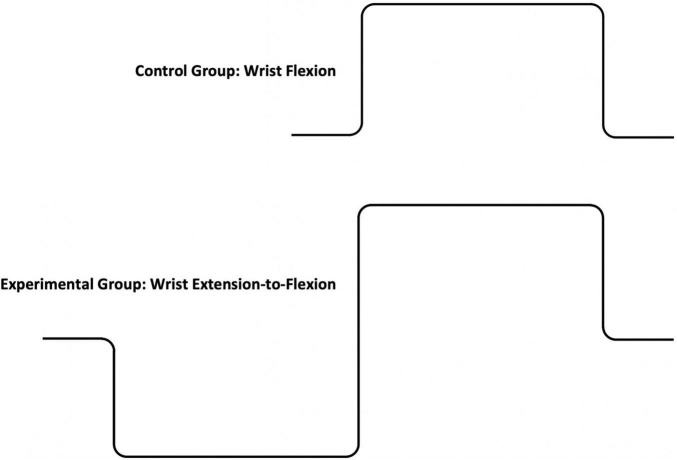
The ideal torque-time curves shown to participants in the control group (top panel) and the experimental group (bottom panel).

Participants were instructed to isolate the action of their forearm muscles and minimize any extraneous movements. Visual feedback was provided using an oscilloscope (Hitachi, VC-6525). The instructions were to contract “as hard and fast as possible,” moving the oscilloscope trace toward a target line representing their maximum torque, and to maintain the trace as close as possible to the target line. The task requirements were reinforced by showing participants a picture of the “ideal” torque-time curve for their assigned condition (Howell, 1956). The ideal torque-time curves for both the control and experimental groups were designed to create a skill requirement, which normally exists for a resistive exercise task (see Figure 1). The key features for performance were how closely the trace on the oscilloscope matched the steepness of the rise of the torque-time curve, and to increase the height of the curve, while maintaining a constant level of torque during the plateau. A graphic of the ideal torque time curve (see Figure 1) was placed above the oscilloscope to reinforce the requirements of the task throughout each test session, except on session 4. Visual feedback was removed during retention testing (session 4). There is a distinction between improvements in performance driven by feedback, where participants are engaged in the trial and error comparison process (acquisition phase: sessions 1–3) versus performance of the task in the absence of any feedback, which occurs after a period of time to allow for consolidation of what was learned and retained from the trial and error comparison process (retention test: session 4) (Lai and Shea, 1999b; Kantak and Winstein, 2012). In the present study, performance was defined by how closely the steepness of the rise of the torque-time curve matched the ideal. The instructions and work-to-rest ratio were controlled by a tape recording. No verbal encouragement was provided during the voluntary contractions.

### Recording Surface Electromyographic Activity

Prior to testing, the electrode locations were shaved, cleansed with isopropyl alcohol, and lightly abraded (NuPrep^®^, Weaver and Company, Aurora, CO, United States) to maintain skin-electrode impedance below 10 kΩ (Grass EZM Electrode Impedance Meter, Astro-Med, Inc., Warwick, RI, United States). The motor points of the flexor carpi radialis (FCR) and extensor carpi radialis (ECR) were located using low-level repeated electrical stimulation on the skin’s surface. The electrodes were then affixed with two-sided tape and electrolyte gel (Signa Gel^®^, Parker Laboratories, Fairfield, NJ, United States). One electrode was placed directly on the motor point while the second electrode was placed with an interelectrode distance of 1 cm in line with the muscle fibers, as observed by twitches produced during motor point location (McIntosh, 2012; Green et al., 2015). A self-adhesive ground electrode was placed on the back of the hand.

The electrode locations were traced with indelible ink and maintained by the participant, to ensure consistent placement across test sessions. If a participant was unable to maintain their tracings, the motor point was once again electrically located, and the electrodes were placed relative to that same location as described above. These procedures have been shown to result in high intraclass reliability coefficients suitable for documenting surface electromyographic (sEMG) activity obtained over long periods of time (Calder et al., 2005; Calder and Gabriel, 2007; Green et al., 2015).

### Signal Processing

The sEMG signals were amplified (Grass P511, Astro-Med, Inc., Warwick, RI, United States) to maximize the resolution of the 16-bit analog-to-digital converter (PCI-6251, DATAQ Instruments, Akron, OH, United States) and band-passed filtered (3–1,000 Hz). Both force and sEMG signals were digitized at 2,048 Hz (DASYLab, DASYTEC National Instruments, Amherst, NH, United States). The force signal was low-pass filtered (20 Hz, 3 dB) using a 4th order Butterworth digital filter offline in MATLAB (The Mathworks Inc., Natick, MA, United States).

### Data Reduction and Criterion Measures

The following criterion measures were calculated from the torque and sEMG signals: (1) mean maximal isometric wrist flexion toque; (2) the maximal rate of torque development; (3) root-mean-square error variability of the rate of torque versus torque phase-plane; (4) the rate of wrist flexion muscle activation; (5) a coactivation ratio for wrist flexor and extensor sEMG activity; (6) and wrist flexor electromechanical delay. The paragraphs below describe how data reduction was completed. The criterion measures were calculated using MATLAB software (The Mathworks Inc., Natick, MA, United States).

**FIGURE 2:**
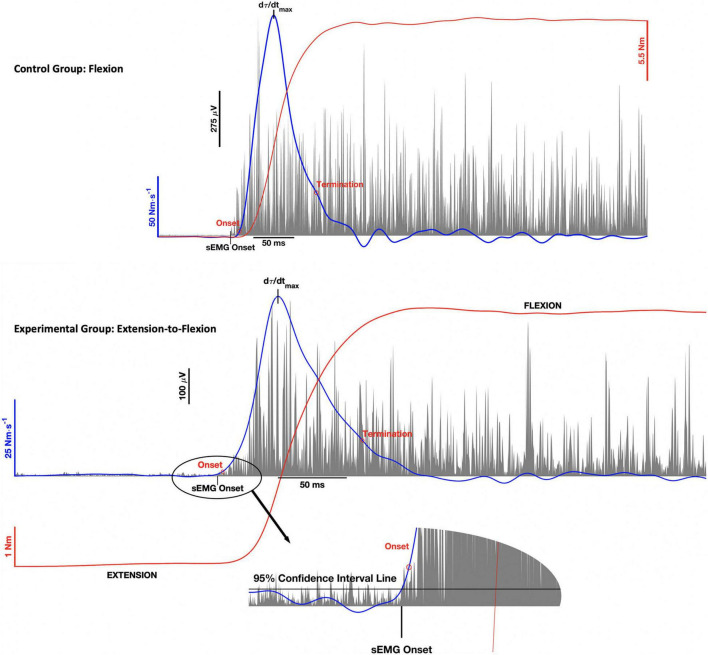
Representative wrist torque (red), rate of torque (blue), and surface electromyographic (sEMG) activity (gray) for the control group (top panel) and experimental group (bottom panel). The graphs illustrate the onset and termination of the torque (τ) development phase of the contraction, where the maximum rate of torque development (*d*τ/*dt*_*max*_) was the peak of the curve between these two points. The double threshold algorithm used to detect sEMG onset was the same for both the control and experimental groups. The figure inset associated with the bottom panel illustrates how the algorithm was applied to the PNF contraction pattern.

Mean maximal torque was taken from the middle of the contraction to ensure that participants had achieved a stable plateau. The rate of torque development was then derived from the torque-time curve using a 10 ms moving window, least squares regression (Lanczos, 1988; Andersen and Aagaard, 2006). The maximum rate of torque development (*d*τ/*dt*_*max*_) was the taken from the peak of the differentiated torque-time curve. Onset of the torque development phase was defined as the first point to exceed 1% of the *d*τ/*dt*_*max*_ and termination was the first point below 20% of *d*τ/*dt*_*max*_ after reaching its maximum (Gabriel et al., 2001b). The onset and termination points are depicted in the top and bottom panels of Figure 2, for the control group and experimental groups, respectively.

**FIGURE 3:**
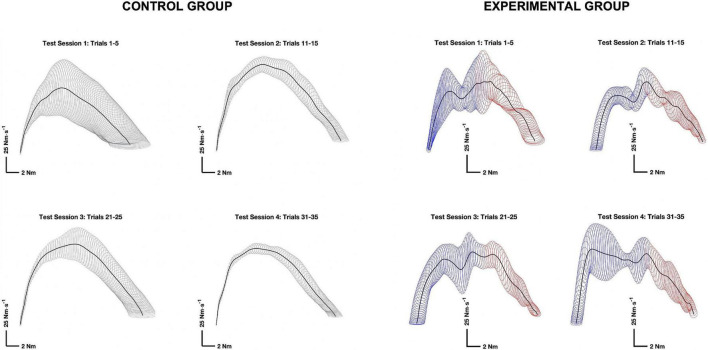
Representative phase plane trajectories for the control (left) and experimental (right) groups. Phase plane trajectories (thick dark lines) were constructed by plotting the rate of torque (*y*) versus torque (*x*), during the torque development phase of the contraction. The thin lines mapped onto the phase plane trajectories are variability ellipses with radii equal to one standard deviation in the rate of torque (*y*) and torque (*x*) directions. Maximal isometric flexion was initiated while the wrist was generating an extension torque and negative. The transition point between the negative torque in extension (blue) and a positive toque in flexion (red) in the experimental group is denoted by a transition in the color of the variability ellipses.

Variability of phase-plane trajectories was used to assessed variability of motor output, which is an important indicator of motor learning (Darling and Cooke, 1987; Gabriel, 2002; McGuire et al., 2014). Phase-plane trajectories were constructed by plotting the rate of torque development versus torque during the initial phase of the contraction. The torque development phase was defined in the same way for both the control and experimental groups: starting from the first point to exceed 1% of the *d*τ/*dt*_*max*_ and terminating at the first point below 20% *d*τ/*dt*_*max*_ after reach its maximum. The data were then normalized in time by interpolating the curves to fit within 400 data points (Gabriel, 2002). The variability of phase-plane trajectories was evaluated by calculating the average area of ellipses (standard deviation squared, SD^2^) with radii equal to one standard deviation in the rate of torque development versus torque, at each point in time (Gabriel, 2002). The variability ellipses were calculated for the first five trials of each test session (see Figure 3).

Several measures were used to evaluate learning-related changes in neuromotor control during the torque development phase. The root-mean-square (RMS) amplitude of sEMG activity for the FCR and ECR were calculated from the first point to exceed 1% of the *d*τ/*dt*_*max*_ and terminating at the first point below 20% *d*τ/*dt*_*max*_ after reaching its maximum. Coactivation was then calculated by dividing the RMS amplitude of the ECR by the FCR. Thus, a decrease in this ratio means that there was less coactivation achieved by either an increase in FCR RMS amplitude and/or a decrease in ECR RMS amplitude (Green et al., 2014).

Electromechanical delay was calculated as the time difference between the onsets of FCR sEMG and the rate of torque development, as identified using the double threshold method (Di Fabio, 1987). For the control group (Figure 2, top panel), the onset of FCR sEMG activity was the first data point to remain above the 95% confidence interval for RMS baseline noise for 20 ms. For the experimental group (Figure 2, bottom panel), FCR sEMG onset was voluntary activation that remained greater than the 95% confidence interval for coactivity levels for 20 ms. Thus, EMD for the experimental group reflects FCR transition from antagonist coactivity to voluntary activation to initiate isometric wrist flexion (see Figure 2, bottom inset).

The rate of increase in FCR muscle activation was calculated by numerically integrating linear envelope detected (60 Hz) sEMG activity, from the onset of torque development to the first 30 ms (*Q*_30_). This definition is different from starting integration from the onset of sEMG (Inglis et al., 2013, 2017). The reason is that the onset of FCR sEMG activity to initiate maximal isometric wrist flexion for the experimental group, occurs during maximal isometric wrist extension. Until the torque-time curve becomes positive indicating flexion, the FCR is momentarily undergoing a quasi-eccentric contraction (Ito et al., 1998; Simoneau et al., 2012), which is known to alter the sEMG-to-force relationship (Babault et al., 2001; Grabiner and Owings, 2003; Pasquet et al., 2005).

To compare *Q*_*30*_ between the two groups, the onset of flexion torque for the experimental group was redefined only for this measure. The onset of flexion torque was defined as the first positive data point of the torque-time curve, after the extension phase of the PNF contraction sequence. The point at which the torque-time curve crosses zero can more easily be seen in the bottom inset of Figure 2. Definition of the onset of wrist flexion torque remained unchanged for the control group. Selecting the onset of wrist flexion torque, did not alter the ability to monitor changes in rate of muscle activation, as motor unit studies have demonstrated alterations in recruitment and rate-coding during this exact time period (Van Cutsem et al., 1998; Del Vecchio et al., 2019b; Inglis and Gabriel, 2020; Kirk and Rice, 2021). In support, preliminary data analysis revealed that the standard and modified definitions for *Q*_*30*_ produced nearly identical results for the control group, differing only in magnitude of the means.

### Statistical Analysis

Previous laboratory data was used to calculate a sample size estimation (*N* = 10) to detect significant differences in the maximal rate of torque development at the 0.05 probability level with a power of 0.80. We collected an additional 3 per group to safeguard against dropout or experimental recording issues. Only one participant failed to complete the experimental protocol, resulting in an unbalanced design, with thirteen subjects in the control group (*N* = 13) and twelve subjects in the experimental group (*N* = 12). A balance was required between using a sufficient number of trials to reliably document changes during the acquisition and retention phases while avoiding fatigue. Preliminary reliability analysis revealed that the first five trials met these criteria. As a result, only the first five trials of each test session were analyzed. There were no significant main effects for either the Group × Trial or Session × Trial interaction terms, so the first five trials were averaged for further hypothesis testing.

An unbalanced split-plot factorial (SPF*p.q*) analysis of variance (ANOVA) with one between groups factor (*p* = flexion-only versus extension-to-flexion) and one within-groups factor (*q* = session) was used to evaluate significant differences. Planned comparisons using orthogonal contrasts were used to document changes in wrist flexion torque measures and sEMG activity at the end of the acquisition phase (session 1 versus 3) and during the retention test (session 1 versus 4) (Lai and Shea, 1999a, b). Effects sizes for planned comparisons for between groups designs was conducted as outlined by Trigo and Martínex (2016) and implemented using the SAS (SAS Institute Inc., Cary, NC, United States) software macro language (Rodriguez de Gil et al., 2013). Interpretation of effect size was based on Cohen’s (1988) benchmarks where *η*^2^ = 0.01 is a small, *η*^2^ = 0.06 is a medium, and *η*^2^ = 0.14 is a large effect size.

## Results

The means and standard deviations for the participant characteristics are presented in Table 1. The control and experimental groups were nearly identical in height, weight, age and in anthropometrics of the limb that was tested. The means, standard deviations, and *F*-ratios for the criterion measures are presented in Tables 2, 3. There was no significant difference between groups with respect to maximal wrist flexion torque. The control and experimental groups exhibited a progressive increase in strength across test sessions. Compared to session 1, the acquisition phase resulted in a 19.57% increase (*p* < 0.001, *η*^2^ = 0.729), which continued over the 2-week rest interval for a total increase of 23.35% on test session 4 (*p* < 0.001, *η*^2^ = 0.655). Changes in the maximum rate of torque development mirrored alterations in wrist flexion strength. That is, there were no significant differences between groups. Contrasting test session 1 versus 3, the acquisition phase resulted in an increase of 19.84% (*p* < 0.001, *η*^2^ = 0.384). There was still a 16.44% increase maximum rate of torque development compared to test session 1 (*p* = 0.076, *η*^2^ = 0.150).

**TABLE 1:** Means (M) and standard deviations (SD) for the physical characteristics of the participants by group.

**Physical characteristic**	**Control group (*N* = 13) M (SD)**	**Experimental group (*N* = 12) M (SD)**
Age (years)	23.47(2.22)	23.61(2.43)
Height (cm)	178.9(6.18)	178.8(5.59)
Weight (kg)	78.08(9.12)	78.38(8.55)
Forearm length (cm)	29.06(1.37)	29.59(1.57)
Forearm circumference (cm)	27.68(1.10)	27.79(1.38)
Hand length (cm)	20.12(0.77)	20.09(1.42)

*No significant differences between groups.*

**TABLE 2:** The means (M), standard deviations (SD), *F*-ratios, degrees of freedom (*df*) and probabilities (*P*), and η^2^effect sizes for maximum wrist flexion torque (τ_*max*_), the maximum rate of torque development (*d*τ/*dt*_*max*_), and the root-mean-square error (RMSE) of the phase plane trajectories calculated from ANOVAs with planned comparisons (ψ).

		**τ_*max*_(*Nm*)**				***d*τ/*dt_max_*(*Nm*⋅*s*^−1^)**				***RMSE* (*SD*^2^)**			
**Session**		**Control**		**Experimental**		**Control**		**Experimental**		**Control**		**Experimental**	
		**M (SD)**		**M (SD)**		**M (SD)**		**M (SD)**		**M (SD)**		**M (SD)**	
1		13.86 (5.40)		12.07 (4.52)		84.21 (36.59)		93.51 (42.80)		3527.25 (1633.65)		3765.48 (1758.17)	
2		16.91 (5.51)		13.98 (5.11)		106.16 (59.82)		96.47 (41.53)		2628.04 (1363.06)		3139.24 (1223.41)	
3		17.65 (5.90)		15.34 (5.00)		104.84 (54.73)		116.89 (58.65)		2726.15 (1148.88)		3706.64 (2999.56)	
4		18.50 (6.80)		16.07 (6.17)		103.85 (57.16)		108.57 (54.61)		2430.83 (833.37)		3571.49 (1546.62)	
**Grand M (SD)**		**16.73 (6.02)**		**14.52 (5.23)**		**99.77 (52.09)**		**103.86 (49.25)**		**2828.067 (1310.26)**		**3545.71 (1949.53)**	
				
**ANOVA F-Ratios**	**df**	***F***	***P***		**η^2^**	***F***	***P***		**η^2^**	***F***	***P***		**η^2^**

**Group**	**[1,23]**	1.12	0.300		0.037	0.08	0.781		0.003	1.88	0.184		0.046
**Session**	**[3,69]**	12.70	<0.001		0.072	3.87	0.013		0.023	2.52	0.065		0.032
**ψ_1,3_**	**[1,23]**	16.77	<0.001		0.729	8.84	<0.001		0.384	318	0.087		0.138
**ψ_1,4_**	**[1,23]**	15.06	<0.001		0.655	3.45	0.076		0.150	4.56	0.044		0.198
**Group × Session**	**[3,69]**	0.59	0.626		0.003	0.86	0.464		0.005	0.62	0.606		0.012

**TABLE 3:** The means (M), standard deviations (SD), and *F*-ratios, degrees of freedom (*df*) and probabilities (*P*) and η^2^ effect sizes for maximum rate of increase in flexor carpi radialis muscle activation (*Q*_30_), the coactivation ratio, and electromechanical delay (EMD) calculated from ANOVAs with planned comparisons (ψ).

		***Q*_30_(*mV*⋅*s*^−1^)**				***Coactivation Ratio***				***EMD (ms)***			
**Session**		**Control**		**Experimental**		**Control**		**Experimental**		**Control**		**Experimental**	
		**M (SD)**		**M (SD)**		**M (SD)**		**M (SD)**		**M (SD)**		**M (SD)**	
1		13.37 (6.17)		13.38 (10.04)		0.28 (0.17)		0.54 (0.37)		32.38 (13.21)		150.71 (69.04)	
2		13.73 (8.40)		15.41 (10.79)		0.27 (0.20)		0.45 (0.38)		31.50 (14.33)		144.42 (32.19)	
3		13.88 (10.30)		12.53 (8.44)		0.26 (0.16)		0.29 (0.15)		27.43 (15.97)		124.52 (28.46)	
4		14.42 (10.74)		12.5 (7.29)		0.26 (0.18)		0.29 (0.23)		30.51 (13.62)		142.25 (45.09)	
**Grand M (SD)**		**13.99 (8.82)**		**13.46 (9.02)**		**0.27 (0.17)**		**0.39 (0.31)**		**30.45 (14.02)**		**140.47 (46.04)**	
				
**ANOVA F-Ratios**	**df**	***F***	***P***		**η^2^**	***F***	***P***		**η^2^**	***F***	***P***		**η^2^**

**Group**	**[1,23]**	0.06	0.808		0.002	3.59	0.071		0.061	154.03	<0.001		0.809
**Session**	**[3,69]**	0.48	0.693		0.004	2.69	0.053		0.057	2.45	0.071		0.351
**ψ_1,3_**	**[1,23]**	0.29	0.595		0.013	6.23	0.020		0.271	4.34	0.049		0.189
**ψ_1,4_**	**[1,23]**	0.06	0.807		0.003	6.12	0.021		0.266	0.36	0.552		0.016
**Group × Session**	**[3,69]**	0.41	0.743		0.003	1.84	0.146		0.039	0.20	0.898		0.003

There was no significant difference in RMSE between Groups (*p* = 0.182, *η^2^* = 0.046), nor was there a significant Group × Session interaction term (*p* = 0.606, *η^2^* = 0.012). The means for both groups alternated between decreases and increases across test sessions, achieving the lowest RMSE on test session 4, after the 2-week rest interval. There was 12.22% (*p* = 0.087, *η*^2^ = 0.138) reduction in RMSE on test session 3 that continued 2-week later for a total of 18.21% (*p* = 0.044, *η*^2^ = 0.198) on test session 4. The alternating pattern can be observed in the representative phase plane trajectories for both groups depicted in Figure 3.

The rate of muscle activation (*Q*_30_) was nearly identical between groups (*p* = 0.808, *η*^2^ = 0.002), and there was no significant difference in *Q*_*30*_ across test session (*p* = 0.693, *η*^2^ = 0.004). Across all four test sessions, the experimental group had a 31.86% greater level of coactivation than did the control group (*p* = 0.071, *η*^2^ = 0.061). However, inspection of the means in Table 3 shows that both groups exhibited a reduction in coactivation during the acquisition phase, and it was retained over the 2-week period on session 4. Contrasting test sessions 1 versus 3, the acquisition phase resulted in a reduction of 32.56% (*p* = 0.020, *η*^2^ = 0.271). The reduction in coactivation was completely retained, as a 32.60% decrease was still observed on test session 4 (*p* = 0.021, *η*^2^ = 0.266). Across all four test sessions, the experimental group had a 77.43% longer EMD than observed for the control group (*p* < 0.001, *η*^2^ = 0.809). Orthogonal contrasts between test sessions 1 versus 3 and 4, showed a 16.82% reduction on test session 3 (*p* = 0.049, *η*^2^ = 0.189) that dissipated to 5.48% over the 2-week retention interval (*p* = 0.552, *η*^2^ = 0.016).

## Discussion

The present study examined the cost-benefit of increasing resistive exercise task complexity to elicit proprioceptive neuromuscular facilitation of the maximal rate of torque development. There were two specific aims. First, it was important to determine if the PNF (extension-to-flexion) contraction pattern at the wrist can result in a greater maximal rate of torque development than simple contractions of the wrist flexors alone. If the PNF contraction pattern can augment the maximal rate of torque development, does the mechanism involve neuromuscular responses or musculoskeletal biomechanics as determine by the presence or absence of concomitant alterations in sEMG activity, respectively. Second, it was necessary to establish if the increase in task complexity associated with the PNF contraction pattern, alternatively interferes with increases in the maximal rate of torque development due to added task complexity during resistive exercise task learning. An increase in the maximal rate of torque development, across test sessions, associated with changes in sEMG activity consistent with improved coordination, would establish that it does not interfere with motor skill learning of the resistance exercise task. In the following paragraphs, we will discuss the present findings.

### Proprioceptive Neuromuscular Facilitation

In the present study, we expected that a maximal isometric contraction of the wrist extensors would facilitate a maximal isometric contraction of the wrist flexors, if performed immediately in succession. The extensor Golgi tendon organs activate the 1b afferents causing autogenic inhibition of the extensor motoneuron pool and facilitation of the flexor motoneuron pool (Moore and Kukulka, 1991). If at that very moment, there is a voluntary command to activate the wrist flexors, central drive and proprioceptive feedback then combine to recruit high threshold flexor motoneurons to augment the flexion contraction (Kabat, 1950; Levine and Kabat, 1953). Despite the basic spinal mechanisms underlying PNF being operative during voluntary contractions (Moore and Kukulka, 1991; Etnyre and Kinugasa, 2002), increases in muscle activation have yet to be demonstrated.

The study by Shimura and Kasai (2002) suggested that PNF may lower motor unit recruitment threshold. Indwelling recordings of motor unit recruitment threshold versus discharge rate by Kirk and Rice (2021) show that alterations in excitability can result in more motor units recruited at lower thresholds, which would increase rate of muscle activation and reduce EMD (Del Vecchio et al., 2019b; Dideriksen et al., 2020). The results for both *Q*_*30*_ and EMD suggest that the PNF contraction sequence did not result in segmental facilitation. There was no significant difference in *Q*_*30*_ between groups, and the EMD was actually longer during the PNF contraction sequence. Thus, the present sEMG findings conflict with the observations of Kamimura et al. (2009) and Richartz et al. (2010) who reported increases in activation.

Kamimura et al. (2009) differentiated linear envelope detected sEMG activity and reported distinct increases in the rate of muscle activation as assessed by *dE*/*dt*_*max*_, and Richartz et al. (2010) reported a dramatic increase in the average magnitude of sEMG from the onset of dorsiflexion sEMG to the first 25 ms of activity, with the reversal contractions. The experimental design and methodological controls of both studies were excellent, and the results cannot be easily dismissed. As is commonly the case when comparing sEMG results, differences in signal processing may, in part, explain the discrepant findings. Both investigative groups made specific assumptions with regards to normalization and corrections for the sEMG-to-force relationships that we have previous shown to alter interpretation of the signal (Inglis et al., 2013; Green et al., 2015).

However, the reason why the findings of Kamimura et al. (2009) and Richartz et al. (2010), cannot be dismissed, is that the present study also failed to corroborate increases in the maximal rate of torque development during a PNF contraction sequence (Gabriel et al., 2001a; Kamimura et al., 2009; Richartz et al., 2010). We believe that muscle mechanics may, once again, explain the discrepant findings. In the case of the upper limb and lower leg, the first contraction of the PNF sequence, stretches the target muscles while coactive as antagonists, placing them at a more optimal length, immediately prior to their voluntary activation as agonists (Blazevich et al., 2009; Simoneau et al., 2012). In contrast, optimal length for the wrist flexors occurs at more flexed wrist angles (Delp et al., 1996; Gonzalez et al., 1997). Wrist extensor activation during the preceding isometric contraction would require the wrist flexors to be coactivated for joint stabilization (Blazevich et al., 2009; Simoneau et al., 2012). The flexors would become taught, placing them at a slightly less optimal, greater length, immediately prior to their voluntary activation during wrist flexion (Hallbeck, 1994; Delp et al., 1996; Gonzalez et al., 1997).

### Motor Learning

The present work corroborates the earlier findings of Gabriel et al. (2001a) who showed that the PNF contraction sequence allows for motor skill related increases in the peak rate of torque development. The present study demonstrated a progressive increase in the rate of maximal isometric torque during the acquisition phase of testing (sessions 1–3). The increase was then retained over the 2-week rest interval on session 4, when any gains associated with physiological adaptations due to a limited number of contractions would have dissipated over the 2-week interval (Häkkinen and Komi, 1983; Mujika and Padilla, 2001). Because task complexity was greater for the PNF contraction sequence, it is not surprising that the variability of phase-plane trajectories was greater than for simple contractions of the wrist flexors (Norrie, 1967; Kasai and Seki, 1992). Nevertheless, the acquisition phase still resulted in an overall decrease in variability for both groups that was retained over the 2-week rest interval. Taken together, the increase in the maximal rate of torque development and the reduction in variability of the phase plane trajectories, both retained on test session 4, indicate that motor learning had occurred (Kohl and Guadagnoli, 1996; Lai and Shea, 1999b; Krakauer and Mazzoni, 2011; Kantak and Winstein, 2012).

Participants did not have any direct knowledge of results related to the maximum rate of torque development, but they were instructed to contract “as hard and as fast as possible” and had visual feedback through the oscilloscope placed in front of them. That is, participants could evaluate the steepness of ascent for the torque-time curve. The constant sensorimotor integration of visual feedback would tend to drive progressive changes in the maximum rate of torque development as previously observed (Lai and Shea, 1999b; Cohen et al., 2001; Rosenkranz and Rothwell, 2012). In contrast, there was no feedback about variability of the torque-time profiles. Rather, the decrease in variability of the phase-plane trajectories was an emergent characteristic of motor learning, that followed a more complex pattern of adaptation (see Figure 3). The fluctuating means across test session are consistent with ongoing development of an internal model of task performance that was updated, refined, and consolidated across test sessions (Shea et al., 2000).

It is somewhat surprising that there was no significant difference in the rate of muscle activation across test sessions, as has been previously observed (Van Cutsem et al., 1998; Barry et al., 2005; Inglis et al., 2017). There are two possible factors that interact to explain the present results. First, changes in the rate of muscle activation due to progressive resistive exercise involve chronic adaptations within the neuromuscular system, which is very different from the limited number of contractions in the present study (Carroll et al., 2001; Gabriel et al., 2006; Del Vecchio et al., 2019a; Siddique et al., 2020; Škarabot et al., 2021). Second, studies that demonstrated changes in the rate of muscle activation due to motor learning, did so in muscles that have a broader motor unit recruitment range (∼80% MVC) such as the biceps brachii (Christie et al., 2009) and the tibialis anterior (Feiereisen et al., 1997), compared to the narrower range (∼50% MVC) of the FCR (Mallette et al., 2018, 2021).

Muscles with a broad recruitment range rely on motor unit recruitment, which can result in more dramatic increases in the slope of the sEMG signal (Van Cutsem et al., 1998; Del Vecchio et al., 2019b; Dideriksen et al., 2020). Muscles with a narrow recruitment range like the FCR, rely primarily on rate-coding for the gradation of muscle force (Kukulka and Clamann, 1981; Seki and Narusawa, 1996). Of course, the rate of FCR muscle activation can increase with the intensity of contraction (Hoffman and Strick, 1993). However, compared to the ankle or elbow, the wrist is primarily associated with static postures involved in object manipulation, where the control strategy depends on regulating joint stiffness, through the coactivation of muscles surrounding the joint (Milner et al., 1995; Werremeyer and Cole, 1997; Schieber and Santello, 2004; Salonikidis et al., 2011; Behrens et al., 2019). In the present study, we observed increases in the maximal rate of torque development in association with a reduction in coactivation, which is consistent with stiffness regulation at the wrist joint (Milner et al., 1995; Kornecki et al., 2001). Moreover, the reductions in both variability of phase-plane trajectories and in coactivation followed a similar pattern of change across test sessions. Thus, task specific adaptations associated with motor skill learning may therefore be governed by the degree to which the joint is involved in stiffness regulation as part of activities of daily living (Hoshizaki and Massey, 1986; Misner et al., 1990; Beveridge et al., 2020).

Electromechanical delay has been observed to decrease with increases in the rate of muscle activation associated with motor-skill learning of maximal effort contractions (Inglis et al., 2017), and has been linked with changes in motor unit activity (Van Cutsem et al., 1998; Van Cutsem and Duchateau, 2005; Del Vecchio et al., 2019b; Kirk and Rice, 2021). It is reasonable to suggest that the decrease in EMD observed in the present study, was indicative of alterations in rate coding due to motor skill learning, but the magnitude of change was not sufficient to detect changes in the rate of muscle activation (*Q*_30_), as would occur in a muscle with a larger recruitment range and/or following progressive resistive exercise training. It is also possible that the maximal isometric contractions performed within a 5-day period during the acquisition phase, induced a short-term increase in stiffness of the muscle-tendon unit that would decrease EMD by test session 3, but would be completely dissipated over the 2-week rest interval (Grosset et al., 2008; Stock et al., 2016; Kubo et al., 2017).

## Conclusion

The wrist extension-to-flexion contraction pattern did not result in a greater maximal rate of torque development than simple contractions of the wrist flexors. The absence of PNF during isometric contractions of the wrist flexors suggests that efficacy may depend on musculoskeletal biomechanics and not segmental facilitation. There was no difference between groups with respect to motor skill learning. The maximal rate of torque development exhibited progressive increases during the acquisition phase and was retained over a 2-week rest interval. The main adaptation in neuromotor control was a decrease in coactivation, not the maximal rate of muscle activation. Resistive exercise task specific adaptations associated with motor skill learning may therefore depend on the degree to which the joint is involved in stiffness regulation as part of activities of daily living.

## Data Availability Statement

The raw data supporting the conclusions of this article will be made available by the authors, without undue reservation.

## Ethics Statement

The studies involving human participants were reviewed and approved by the Brock University Research Ethics Board (REB#12-281). The patients/participants provided their written informed consent to participate in this study.

## Author Contributions

LG and JM participated in the data collection. DG completed the data reduction. All authors contributed equally to the data analysis, interpretation of the results, and preparation of the manuscript.

## Conflict of Interest

The authors declare that the research was conducted in the absence of any commercial or financial relationships that could be construed as a potential conflict of interest.

## Publisher’s Note

All claims expressed in this article are solely those of the authors and do not necessarily represent those of their affiliated organizations, or those of the publisher, the editors and the reviewers. Any product that may be evaluated in this article, or claim that may be made by its manufacturer, is not guaranteed or endorsed by the publisher.
